# Patent ductus venosus in an infant with direct hyperbilirubinemia

**DOI:** 10.1002/ccr3.2266

**Published:** 2019-06-14

**Authors:** Leila Kamali, Maryam Moradi, Shadi Ebrahimian, Mahsa Masjedi Esfahani, Mohamad Saleh Jafarpishe

**Affiliations:** ^1^ Department of Radiology Isfahan University of Medical Sciences Isfahan Iran

**Keywords:** hyperbilirubinemia, jaundice, patent ductus venosus, portosystemic venous shunt

## Abstract

Patent ductus venosus is caused by a defect in obliteration of ductus venosus after birth. Ductus venosus connects umbilical vein and inferior vena cava during fetal period. Patent ductus venosus is a very rare cause of cholestatic jaundice.

## 1 INTRODUCTION

Ductus venosus is a connection between portal vein and inferior vena cava that closes functionally in first minutes after birth and true obliteration is completed in 15‐20 days and ligamentum venosum is formed.^1^ But, closure of ductus venosus may not happen after birth because of poorly developed intrahepatic portal system and lead to persistent ductus venosus (PDV).^2^ PDV is a rare disease and results in diversion of portal blood into the systemic circulation and is a type of congenital portosystemic shunt.^3^

## 2 CASE HISTORY

The patient was a 69‐day‐old breastfed male infant with a gestational age of 39 weeks. His birth weight was 2700 g, height 49 cm, and head circumference 38.5 cm, following a nonconsanguineous marriage and a normal vaginal delivery from a 32‐year‐old mother (gravida 3, para 3) with a well‐controlled gestational diabetes mellitus during pregnancy. Prenatal screening tests were done completely, and no other abnormality was detected.

The patient was referred to Imam Hossein hospital, located in Isfahan city, Iran, for further evaluation of prolonged direct hyperbilirubinemia and cholestatic jaundice which was noted by parents at age of 25 days after birth. When he was admitted at age of 27 days, he was afebrile and no tachycardia and tachypnea were present. He was not ill and his general appearance was normal, no bruising or petechiae was seen. No abnormality was detected in funduscopic, cardiac, or abdominal examination. No skeletal abnormality, dysmorphia, or deformity was detected. The patient had a normal neurological examination and psychomotor development.

The patient was evaluated because of conjugated jaundice. In the first assessments, urine analysis and culture were normal. No thrombocytopenia was detected. Metabolic screen tests and sweat test were unremarkable, and serum alpha‐1 antitrypsin was within normal range (103.1 mg/dL). Alpha‐fetoprotein level was normal for age.

During evaluations, a mildly elevated thyroid function tests were detected (6.78 mic/IU/mL) for which he was followed for the possibility of hypothyroidism 1 month later and levothyroxine was started because of a rise in TSH (14 mic/IU/mL) (Table 1).

**Table 1:** Progressive improvement of liver function tests after embolization

	At admission time	10 wk after fistula embolization	19 wk after fistula embolization	Normal ranges
Total Bilirubin	10.9 mg/dL	0.7 mg/dL	0.6 mg/dL	0.1‐1.1 mg/dL
Direct Bilirubin	6.8 mg/dL	0.2 mg/dL	0.1 mg/dL	0.1‐0.3 mg/dL
ALT	167 U/L	207 U/L	65 U/L	Up to 40 U/L
AST	530 U/L	119 U/L	51 U/L	Up to 43 U/L
ALKP	2305 U/L	812 U/L	933 U/L	1000‐1076 U/L
PTT	35.00 s	29.00 s	‐	28‐40 s
PT	11.00 s	10.30 s	‐	10‐12 s
Alpha‐fetoprotein	>1210 ng/mL	62.1 ng/mL	‐	0‐7 ng/mL

Laboratory examinations revealed direct hyperbilirubinemia, elevated alanine aminotransferase (ALT), aspartate aminotransferase (AST), and alkaline phosphatase with gamma‐glutamyl transferase in normal range (Table 1). A low level of albumin (2.9 g/dL) was detected as an evidence of hepatic dysfunction.

Abdominal ultrasonography was done as the first‐line imaging investigation and revealed significant dilation of left portal vein and shunt of left portal vein into middle hepatic vein which was seen as a cystic dilation measured 13*9 mm without any turbulent flow suggesting PDV between left portal vein and inferior vena cava with secondary hepatic failure.

The patient underwent hepatobiliary scintigraphy with suspicion of biliary atresia resulting in mild to moderate hepatic dysfunction. There was no tracer accumulation in the region of the gallbladder on expected time, and no biliary to bowel clearance was seen even on late 24 hours views.

**Figure 1:**
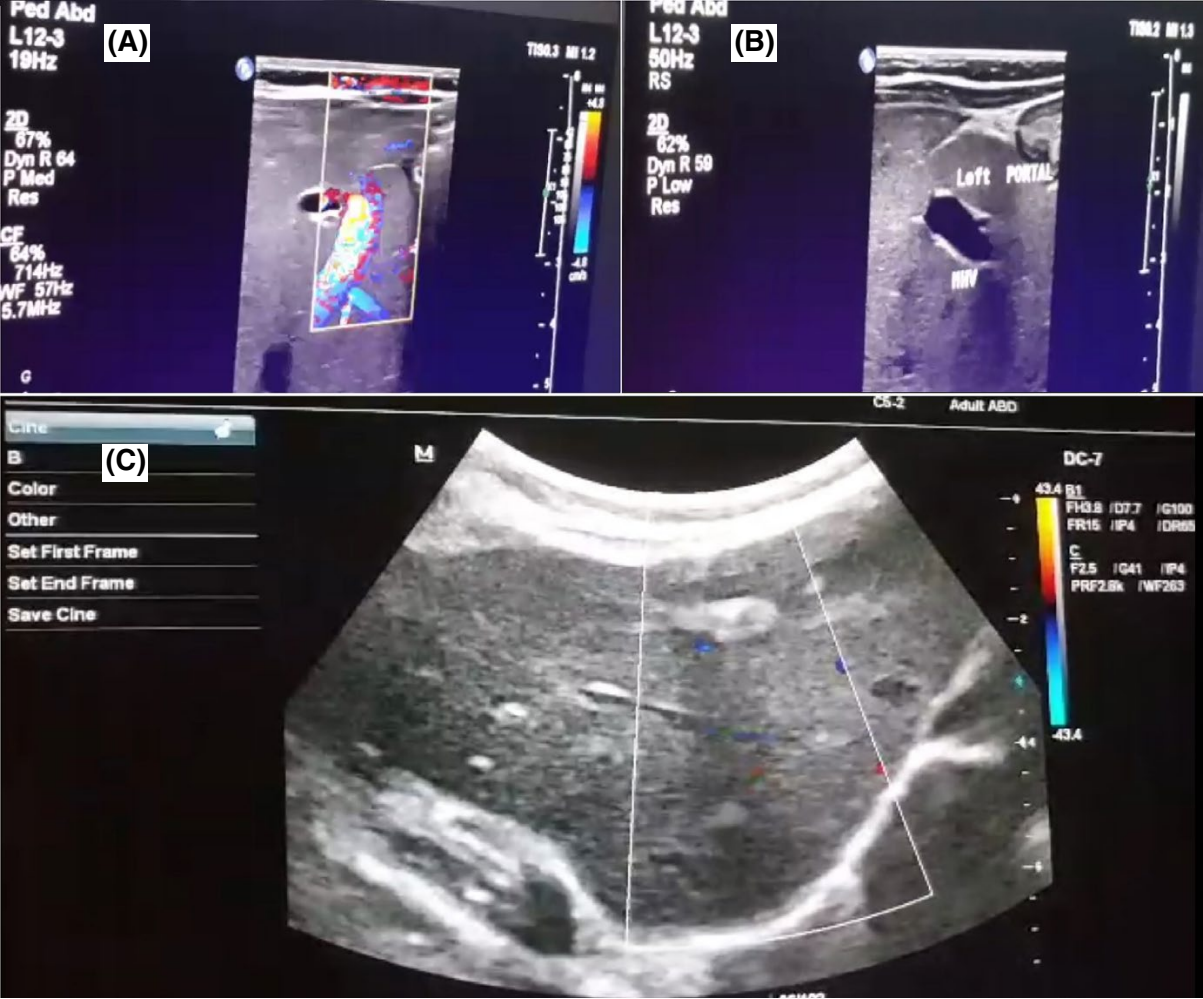
A, color Doppler mode ultrasonography before coil insertion; B, Gray‐ scale ultrasonography before coil insertion; C, Color Doppler mode ultrasonography after coil insertion

**Figure 2:**
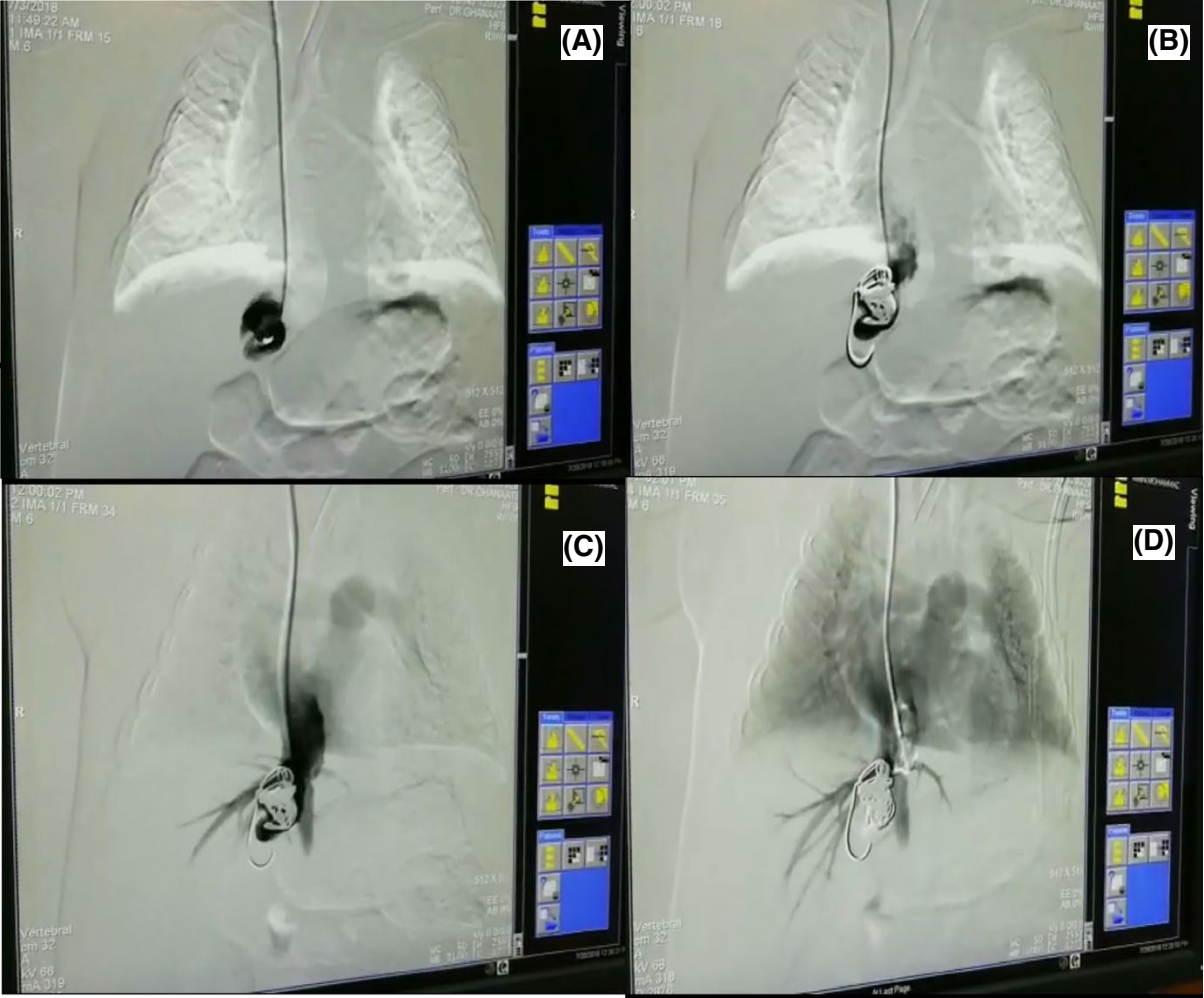
A, Angiography before coil insertion. B‐D, Angiography after coil insertion

Due to the probability of PDV, the CT‐angiogram was done at age of 69 days. During angiography, right atrium, right ventricle, inferior vena cava, and right hepatic vein through jugular vein were entered, a fistula was seen between left hepatic vein and left branch of portal vein. A short hepatic vein was seen and it was directly attached to fistula. Embolization of fistula was done using four biologic coils. Figures 1 and 2 show before and after fistula embolization and coil insertion. The patient was followed up and Bilirubin and liver function tests (LFT) were repeated 10 and 19 weeks after embolization. A decrease in bilirubin and LFTs was seen, and the patient was no more icteric (see Table 1). Ultrasonography was performed and indicated an echogenic area in previous hepatic shunt location. Ductus venosus was completely closed, and no vascular flow was detected indicating proper function of coils.

## 3 DISCUSSION

Liver dysfunction and encephalopathy are important initial presentation of PDV and may be secondary to reduction of blood flow in portal vein which deprives hepatocytes of nutrients and cause liver dysfunction including protein synthesis.^4^

We reported an infant with PDV which developed liver dysfunction and prolonged direct hyperbilirubinemia and low serum albumin which were improved after coil embolization. Liver dysfunction secondary to PDV has been reported previously in three Japanese brothers at ages 3 and 5, and 5 months old^4^ (Table 2). It was also reported in three adult brothers who had hepatic dysfunction symptoms and the same vascular anomaly was detected in ultrasound^5^ (Table 2). The presence of the PDV in family members in previous reports suggested recessive genetic trait of PDV,^5^ this is while we reported a case with no history of PDV in other family members especially in his older siblings. Other cases with no family history of PDV have been reported which makes genetic trait less likely and indicate PDV more likely to be multifactorial, however lacking information about twin studies^6^, ^7^ (Table 2).

**Table 2:** Case's data

Sex	Age at diagnosis	Clinical features	Protocols for diagnosis	Management
M	3 y	Hepatic encephalopathy, hyperammonemia	I‐iodoamphetamine per‐rectal portal scintigraphy	Surgical ligation
M	5 y	Liver failure, hyperammonemia	I‐iodoamphetamine per‐rectal portal scintigraphy	Surgical ligation
M	5 mo	Hypergalactosemia, hyper bile acidemia	Ultrasonography	‐
M	12 y	Hepatic encephalopathy	Doppler abdominal ultrasound, CT scan, abdominal angiography	Surgical ligation
M	Prenatally (Age at surgery: 4 y)	Hepatic encephalopathy, Noonan syndrome	Doppler ultrasound	Surgical ligation
F	3 wk	Cholestatic jaundice, failure to thrive	Abdominal ultrasound, angiography	Detachable vascular plug device
M	43 y	Hypoalbuminemia, pedal edema	Abdominal ultrasound, CT scan, Angiography	‐
M	36 y	Fatigue, pedal edema, intermittent encephalopathy	CT scan, Angiography	‐
M	40 y	Peripheral edema, liver dysfunction	Ultrasound, CT scan	‐

CT, computed tomography; F, female; M, male.

Development of patent ductus venosus and other portosystemic shunts has been associated with prematurity, chromosomal abnormalities including Down syndrome and other malformations including congenital heart disease, pulmonary hypertension, hydrops fetalis, multiple coronary fistula, hypoplastic right hepatoportal system, tumor‐like lesions of the liver, cirrhosis, fatty infiltration of liver, and polysplenia syndrome.^8^, ^9^ In our case, none of the mentioned abnormalities were detected and there were no other risk factors in mother except a well‐controlled gestational diabetes mellitus. The cause of persistency of ductus venosus in this patient remained unknown.

The main complications of portosystemic shunts including PDV are cholestasis of neonates, encephalopathy, hepatopulmonary syndrome, pulmonary hypertension, and liver tumors. In order to prevent these complications, shunts should be closed. Although spontaneous closure may occur after 1‐2 years, other shunts require closure using endovascular or surgical methods.^9^

Diagnostic tool in PDV diagnosis is Doppler ultrasonography detecting vascular formation connecting inferior vena cava and portal vein, and the gold standard is angiography. Laboratory results including hyperammonemia, elevated serum bile acid level, and high level of blood galactose without enzyme deficiency have been reported as important laboratory findings which are suggestive for PDV.^4^ In our case, direct hyperbilirubinemia was lead to diagnosis of PDV and improved after surgical intervention, suggesting that PDV was the cause of direct hyperbilirubinemia. This report and other studies suggest that portosystemic shunt should be considered as a cause of neonatal cholestasis, while other common causes which can be associated with shunt, including biliary atresia should also be considered.^9^, ^10^

While this case reported direct hyperbilirubinemia associated with PDV in an infant, the relation between these findings requires more studies. Another infant with conjugated hyperbilirubinemia and elevated liver enzymes due to PDV was reported in South Africa and was diagnosed using abdominal ultrasonography. The infant was treated by a minimally invasive method using detachable vascular plug device, and a marked improvement was seen 1 week after the procedure.^6^

Persistent ductus venosus also was reported in a 12‐year‐old male with a history of prematurity, intrauterine growth retardation, and dysmorphic features, presented with hepatic encephalopathy and liver mass. He was treated by surgical ligation of PDV.^7^ It was also diagnosed in a male infant who was born premature with Noonan syndrome and went under surgical ligation at age of 4 years old^7^ (Table 2).

Although in our patient a mild hypothyroidism was detected during evaluation of icterus, it did not seem to be the reason for direct hyperbilirubinemia due to lack of improvement in clinical symptoms and laboratory results after starting the treatment.

In an infant of a diabetic mother, the probability of different malformations including neural tube defect, cardiac defect including truncus arteriosus and transposition of great vessels, bilateral renal agenesis, caudal regression, and other vertebral anomalies is higher than the average.^11^, ^12^ In this case report, no other anomalies except PDV were seen in infant of mother with gestational diabetes mellitus, due to a well‐controlled diabetes in mother during pregnancy period.

The treatment of PDV is controversial, and there are different therapeutic methods including conservative management and surgical treatment including coil embolization, Teflon banding, ligation, and liver transplant.^13^ In this case, coil embolization was used and resulted in clinical improvement.

In conclusion, physicians should keep PDV in mind as a rare cause of indirect hyperbilirubinemia in neonates and infants. PDV can be diagnosed by noninvasive imaging studies including ultrasonography. Early detection of the shunt and proper management leads to a good prognosis and prevention of serious complications development.

## CONFLICT OF INTEREST

None declared.

## AUTHOR CONTRIBUTION

MM and MM and LK and SE and MSJ: gathered the patient's data and collected materials. SE: prepared the review and wrote the manuscript. All authors read and approved the final manuscript.
